# Diagnosis of Partial Discharge Based on the Air Components for the 10 kV Air-Insulated Switchgear

**DOI:** 10.3390/s22062395

**Published:** 2022-03-20

**Authors:** Qipeng Tan, Tiandong Zhang, Shaocheng Wu, Jiachen Gao, Bin Song

**Affiliations:** 1 School of Electrical Engineering and Automation, Wuhan University, Wuhan 430072, China; qidipang1224@163.com (Q.T.); ztd1125296686@126.com (T.Z.); 2016302540157@whu.edu.cn (S.W.); jiachen.gao2@unibo.it (J.G.); 2 Department of Electrical, Electronic, and Information Engineering, ‘Guglielmo Marconi’, University of Bologna, 40136 Bologna, Italy

**Keywords:** partial discharge (PD), gas detection, fault classification, random forests, air-insulated switchgear

## Abstract

Partial discharge (PD) is a common phenomenon of insulation aging in air-insulated switchgear and will change the gas composition in the equipment. However, it is still a challenge to diagnose and identify the defect types of PD. This paper conducts enclosed experiments based on gas sensors to obtain the concentration data of the characteristic gases CO, NO_2_, and O_3_ under four typical defects. The random forest algorithm with grid search optimization is used for fault identification to explore a method of identifying defect types through gas concentration. The results show that the gases concentration variations do have statistical characteristics, and the RF algorithm can achieve high accuracy in prediction. The combination of a sensor and a machine learning algorithm provides the gas component analysis method a way to diagnose PD in an air-insulated switchgear.

## 1. Introduction

Based on advanced sensing and measurement technology, two-way communication is an essential feature of the smart grid to ensure reliable and safe operation. In particular, the running status of air-insulated switchgear, critical pieces of equipment in the distribution network, contains critical information about power grid monitoring. During operation, insulation degradation such as internal impurities, bulges, and other defects in the air-insulated switchgear can easily cause a strong electric field area. When the electric field intensity exceeds a certain threshold, it will cause partial discharges (PD) [1]. Regarded as a latent period of insulation deterioration [2], if it is not found and handled in time, PD will evolve into insulation failure. Therefore, PD detection is a critical factor for judging the running state of air-insulated switchgear, playing an important role in constructing the smart distribution network.

According to the phenomenon generated by PD, such as sound, light, and electricity, the primary methods used for PD detection are based on acoustic emission [3,4], pulse current [5], transient earth voltage [6], and ultrahigh frequency [7]. PD detection technology has been used to optimize insulation materials, detect equipment faults, and in other fields [8,9,10]. However, the methods described above are vulnerable to disturbance and are only able to achieve an external measurement. Consequently, to realize live monitoring, many scholars analyze the chemical changes of air molecules caused by PD and study the application of gas composition analysis for their detection. Gas detection is more convenient and efficient, with a more powerful anti-jamming ability than the other methods. Based on the method of gas composition analysis, an integrated sensing system could be established for PD detection.

In the PD process, the dissociation by electron collision is the dominant factor that will cause air decomposition. The new gas molecules will be generated by the collision of a large number of energetic electrons with air-neutralizing molecules. Zhang et al. [11,12,13] analyzed the reactions involving O, C, and N elements and carried out experiments to obtain the concentrations changes of CO and NO_2_. Wang et al. [14,15] determined that CO, NO_2_, and O_3_ can be used as characteristic gases to detect PD through the simulation software Gaussian. However, the existing study focused on finding chemical processes and monitoring the characteristic components instead of classifying concentration data for fault identification.

In PD defect identification based on gas composition analysis, a lot more work has focused on GIS or the power transformer. The typical one is the three-ratio method, which has been used to judge the operation state of transformers. On PD defect diagnosis for GIS, coding recognition was proposed to process the data of SF_6_ decomposition [16]. In recent years, some studies have used machine learning to identify PD defects. Dai employed fuzzy c-means to recognize different kinds of PD by choosing three concentration ratios as feature parameters [17]. The support vector machines (SVM) algorithm combined with the wavelet analysis technique was used to discriminate PD [18]. Based on dissolved gas analysis (DGA), more scholars adopted the artificial neural network (ANN), the polynomial neural network (PNN), etc., [19,20,21]. Moreover, some studies have optimized the algorithms to achieve better results [22,23,24]. Compared to the three-ratio method, the adoption of machine learning significantly improved the accuracy of diagnosis. However, little research has studied PD defect identification for air-insulated switchgear.

Therefore, in order to achieve PD defect identification by analyzing the gas components inside the switchgear, this paper carried out experiments to obtain data and used a machine learning algorithm to complete the classification. CO, NO_2_, and O_3_ were selected as the characteristic gases for detecting PD based on the stability and generation rate of the new gas molecules. To achieve a timely diagnosis, the random forest (RF) algorithm was adopted to explore the possibility of identification. The results showed that the combination of a sensor and machine learning algorithm provided a good way to apply the gas component analysis method to the diagnosis of PD in the switchgear.

## 2. Experimental Setup

**Figure 1:**
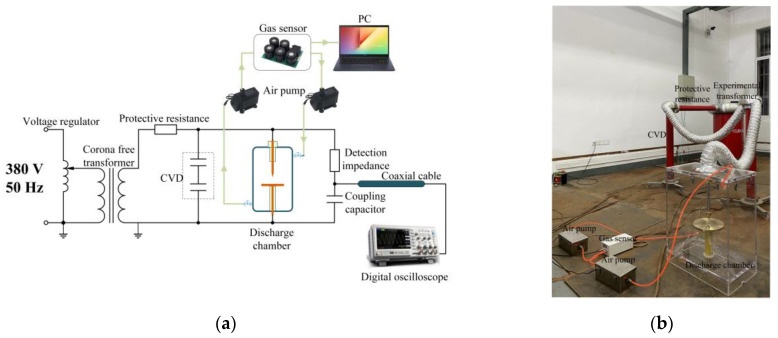
Experiment Platform: (**a**) schematic diagram of the experiment platform; (**b**) layout of the experiment platform.

An experiment platform was built, as shown in Figure 1. The power signal was generated by the combination of the voltage regulator and experimental transformer, which could produce a 50 Hz voltage of up to 50 kV. The gas sensor collected the parameters of characteristic gas. All the measured results were input into the PC.

### 2.1. Setup of Discharge Model

**Figure 2:**
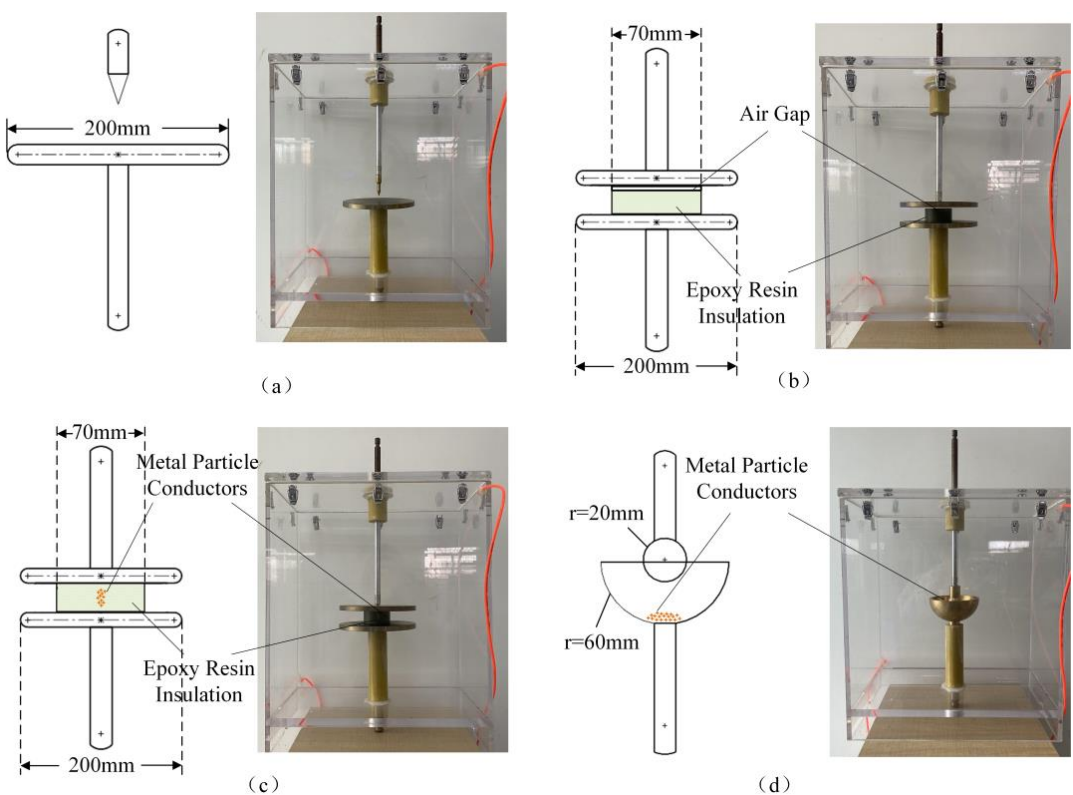
Four types of simulated defects: (**a**) metal protrusion, (**b**) air gap between the metal conductor and the insulator, (**c**) pollution on the insulator surface, and (**d**) charged metal particles.

Four types of simulated defects were built in the discharge chamber. They were metal protrusion, an air gap between the metal conductor and the insulator, pollution on the insulator surface, and charged metal particles, which were sorted out from the analysis of various defects in the air-insulated switchgear. The needle electrode, plate electrode, and ball electrode were all made of copper. The metal particles were also made of copper with a volume of about 1cm^3^. The specific structure and size are shown in Figure 2.

### 2.2. Measuring of Characteristic Gases Concentrations

As shown in the upper right corner of Figure 1a, a set of gas circulation systems was designed by using a gas sensor and a couple of gas pumps. Based on electrochemical detection technology, the gas sensor could be operated simply by computer control and had the advantages of fast response, high accuracy, good stability, and repeatability. Critical components in the sensor included the sampling probe, gas sensor, and unit of data processing, storing, and transmitting. Before beginning the experiment, standard gas was used to calibrate the gas sensor to ensure the data reliability. The unit used to measure the gas concentrations was part per million (ppm), meaning the volume fraction of the characteristic gases as a percentage of the air volume in parts per million

Two gas pumps were set up to extract the mixed gas in the air chamber to the gas sensor and change the gas with the surrounding air before the experiment. They were micromotors with variable power. The flow rate was controlled at 2 L/min, which not only minimized the influence of gas-flowing factors on the discharge decomposition reaction but also met the requirements of the China National Standard System (GB/T15438-1995) for the residence time of air in the gas path system.

## 3. Experiment Results

### 3.1. Partial Discharge

The components were connected according to Figure 1 and grounded safely. Firstly, we continuously increased the voltage until the air gap was broken down and recorded the breakdown voltage. Then, we added voltage until the PD signal could be found on the oscilloscope and maintained the discharge. The applied voltages of the first three defects were about 70% of the breakdown voltages. As for the charged metal particles, it was necessary to apply a voltage close to the breakdown voltage, which would cause the apparent and stable PD phenomenon. Over the next 24 h, the gas sensor collected the concentration data of the characteristic gas every two hours. The voltage applied during the experiment for each defect is shown in Table 1.

### 3.2. The Volume Fraction

**Figure 3:**
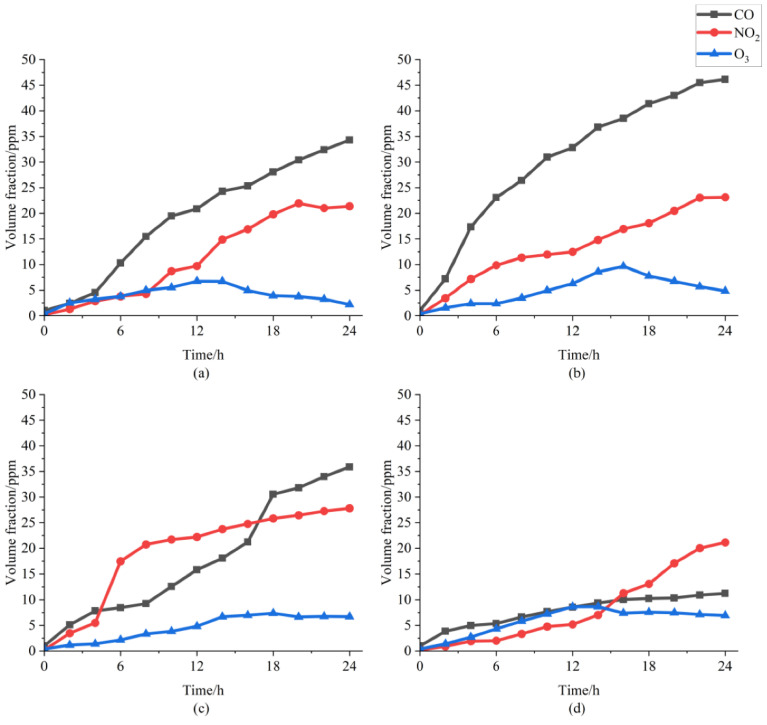
The volume fraction of characteristic gases over time: (**a**) metal protrusion, (**b**) air gap between the metal conductor and the insulator, (**c**) pollution on the insulator surface, and (**d**) charged metal particles.

Five repeated experiments were carried out for each defect to reduce the impact of random error. The growth trends of the characteristic gases over time collected by the gas sensor are displayed in Figure 3.

The volume fraction data indicate that there would be different growth trends and gas production rates of characteristic gas under different discharge modes. The experimental results were not surprising, since the electric field distribution and apparent charge of PD were different among different modes.

The CO concentration in the four different defect models continued to increase. The PD generated by the metal protrusions was mainly due to the electric field distortion at the metal tip, which was persistent and had a corresponding relationship between the number of electrons excited at the same time. However, when the PD was intense, the C elements in the air were not enough to provide continuous excitation. Therefore, the concentration level of CO was low. The CO produced under the air gap between the metal conductor and the insulator was much higher than the other defects. That was because the material of the insulator was epoxy resin with a large amount of C elements. Electrons penetrated the insulation material and continuously stimulated it to release C elements. In the case of pollution on the insulator surface defect, the PD was mainly concentrated on the metal on the insulator surface, so the C element excited was lower than the previous one. As for the defects of charged metal particles, the CO production was the lowest and an obvious saturation phenomenon appeared after 16 h due to the unstable and low discharge.

Furthermore, the NO_2_ concentration changing with time in the first three defects showed a trend of saturation. Due to the spark discharge of the charged metal particles, it produced NO and provide the intermediate products for NO_2_. Moreover, the reason for the trends of O_3_, which exhibited a curve shape of the inverted ‘V,’ may be due to a series of oxidation reactions between O_3_ and NO and CO.

## 4. PD Defect Recognition

PD detection in switchgear based on gas components requires a fast and accurate method. Processed by a specific algorithm, the characteristic gas volume fraction data can be classified and identified to determine the fault type. In this part, 135 groups of the gas concentration data were randomly selected as the sample, of which 100 groups were training samples and 35 groups were test samples. These samples were from the characteristic gas concentrations of different time nodes in different groups of experiments. In 100 groups of training samples, there were 25 groups of each defect type. The data were input into the classification system for defect recognition and analysis.

### 4.1. DT Optimization and RF

RF is a statistical learning theory with fast and high accuracy with expected development prospects. It uses the Bootstrap resampling method to model multiple samples of decision trees (DT) and then combines the results of multiple DT to vote for the final classification result [25].

DT is the basis of RF. It is a tree structure, starting at a root node and ending at the leaf nodes, which can simply and intuitively describe the data classification process. Except for the leaf nodes, other nodes represent splitting attributes of DT. At the end of the branches are the leaf nodes, which correspond to the classification result. When the classification results given by all leaf nodes are the same, the growth ends and the sample class can be determined.

Classification and regression tree (CART) was used to construct a single DT [26], where the *Gini* impurity was recommended to replace information gain as a measure of data impurity. The expression is as follows:

(1) $$Gini\left(D\right)=1-\sum_{i=1}^{m}P_{i}^{2}.$$

In Equation (1), $P_{i}$ represents the probable occurrence of each category in sample set D, which is divided into m categories. 

When the sample set D is split into two subsets, $D_{1}$ and $D_{2}$, the *Gini* impurity is:

(2) $$Gini_{split}\left(D\right)=\frac{\left|D_{1}\right|}{\left|D\right|}\;Gini\left(D_{1}\right)+\frac{\left|D_{2}\right|}{\left|D\right|}\;Gini\left(D_{2}\right).$$

Since the splitting method for each attribute is not unique, the *Gini* impurity after splitting according to a particular attribute has multiple values, of which the minimum is generally used to represent the *Gini* impurity after splitting with the attribute. In addition, the formula of the *Gini* impurity increment is given as:

(3) $$\Delta Gini\left(R\right)=Gini\left(D\right)-Gini_{R}\left(D\right)$$

In Equation (3), R represents the dataset D, split according to an attribute R. When the ΔGini(R) is the largest, the DT has achieved the best performance.

**Figure 4:**
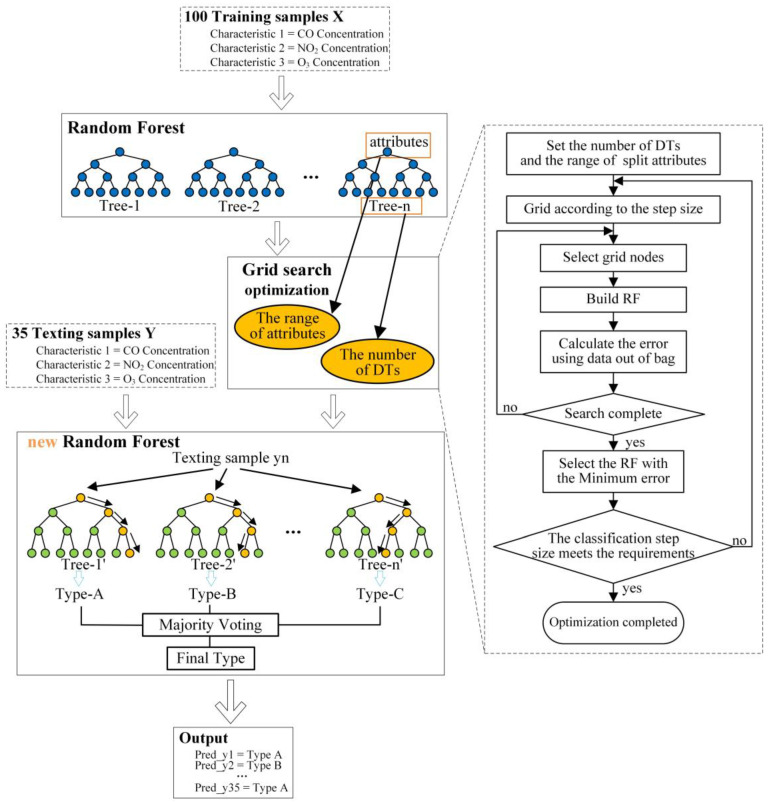
The framework of the algorithm and the optimization process.

The result of the RF is related to the number of splitting attributes of DT. Grid search was used to optimize the algorithm to find the best DT and candidate attributes. The method was to divide the variable area and traverse all grid points to find the optimal parameter value. Firstly, this paper set the scope and step size of the number of DT and candidate attributes. Then a grid was drawn, whose abscissa was the number of DT and ordinate was the number of candidate attributes. Each node in the grid corresponded to a possible RF. After calculating the value of each node, an RF was built. Then the error was calculated according to the out-of-bag data. Comparing the error and choosing the smallest one, its number of DT and candidate attributes was the best if its error met the requirements. If not, the whole process should be repeated. The framework of the algorithm and the optimization process is shown in Figure 4. 

### 4.2. Defect Classification

Before establishing the RF fault diagnosis model, the four defect types in the air-insulated switchgear were numbered, as shown in Table 2.

**Figure 5:**
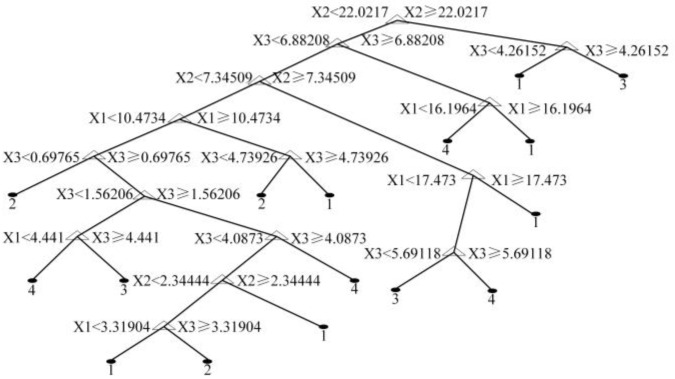
Generation of test set decision tree.

According to the algorithm and optimization process mentioned above, one of the best structures of DT with the minimum *Gini* impurity is shown in Figure 5. The tree, whose attribute of the root node was $x_{2}\langle 22.0217|x_{2}\geq 22.0217$, had the most significant increment ΔGini(R). 

**Figure 6:**
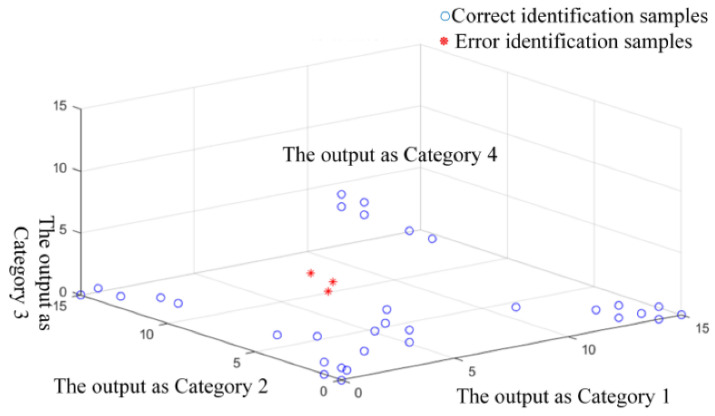
Performance of random forest classifier.

The RF algorithm classified the characteristic gas concentration data based on the above DT optimization. The classification results are shown in Table 3 and Figure 6. As shown in the table, the accuracy was 91.43%, with 32 groups identified correctly. Analyzing the outcome, the identification of pollution on the insulator surface defect was relatively low. This may be because the content of NO_2_ under the defect changed more smoothly, which was likely to be close to the gas concentration data of other defects. At the same time, the different proportions of each defect type in 35 groups of test samples would also affect the final classification results. In addition, to verify the algorithm’s reliability, a threefold cross-validation was carried out on the basis of the same size of training and testing samples. The other two accuracies of the classification results were 94.29% and 88.57%.

## 5. Discussion

To study the online PD diagnosis in 10 kV air-insulated switchgear by gas composition analysis, an experiment platform was set up, and RF was used to identify defect types. In addition, this paper also adopted two basic classifiers, SVM and logistic regression (LR), to compare the results. The confusion matrix of the SVM and LR are shown in Table 4 and Table 5.

The accuracy of the SVM and LR classifiers were 74.29% and 88.57%, respectively, which were both lower than the RF. In particular, the SVM precision of Type 4 was only 57.14%, showing that the SVM classifier easily considered other types as Type 4. Similarly, the LR classifier would consider other types as Type 1, which caused the lower accuracy. In addition, the SVM judged five groups of Type 2 as others. The reason may be that the characteristic gas concentrations of Type 2 for some periods were indistinguishable from Type 1. However, RF made more attribute comparisons between Type 1 and Type 2, which significantly improved the recall rate of Type 2. Because of the steady growth of NO_2_ of Type 3, the three algorithms had errors in its recognition.

In the field of Mathematical Science, RF can deal with multiclass classification well. It has been applied to machine learning to solve the problems of land use, urban population distribution, and disaster prediction. In terms of a power system, there were some examples of using RF to forecast power load [27]. However, it has been rarely used for fault defect classification of power equipment. The identification outcome indicates that the RF algorithm achieved excellent results in identifying gas decomposition characteristics. RF algorithms can process large data faster, achieve higher accuracy, and have better scalability. These outstanding features make it possible to be applied to more areas of electrical equipment fault diagnosis. With the development of computer science and deep learning, using intelligent algorithms to realize fault identification will become a trend for research. Compared with the methods of analyzing the ratio of gases, the RF can overcome the problem of missing code. Moreover, it can avoid the negative impact of unbalanced data compared with the artificial neural network. Accordingly, the extensibility of RF can be applied to more electrical equipment fault identification.

However, there are some limitations of the study. On the one hand, the number of training samples was insufficient due to the long time required for individual experiments. This may cause incorrect recognition results. On the other hand, the algorithm can be further optimized for better identification accuracy. Since the switchgear is used in ambient conditions, there are many factors that can affect the concentration of characteristic gases, such as temperature, humidity, internal structure, or elements of the equipment. The influence of these factors needs to be considered in further research. In a word, the method of gas composition analysis combined with a classification algorithm would provide a reference for PD online monitoring. The ultimate goal of this study is to manufacture a built-in instrument that can detect and transmit PD information in real time for the smart distribution network.

## 6. Conclusions

According to the mechanism of air decomposition, this paper built a PD simulation experiment of a 10 kV air-insulated switchgear and collected the characteristic gas data of four PD defect types. After the experiment, the RF classification algorithm was used to explore the application of gas component analysis in PD defect type identification. The following conclusions were obtained:

(1) Selecting CO, NO_2_, and O_3_ as the characteristic gases of PD can enable effective identification of the defect types.
(2) The experimental results of the characteristic gases volume fraction were consistent with the previous research results of air decomposition products.
(3) The classification system used the RF algorithm to construct the fault recognition model with the minimum *Gini* impurity and achieved excellent results in the identification, with an accuracy up to 91.43%.

The conclusions show that the combination of sensor and machine learning algorithm in the air-insulated switchgear provides way to diagnose PD and a good base for applying the gas component analysis method.

## Figures and Tables

**Table 1:** The voltage applied for each defect.

Type	Metal Protrusion	Air Gap between the Metal Conductor and the Insulator	Pollution on the Insulator Surface	Charged Metal Particles
Applied Voltage (kV)	8	13	8.2	12.8
Breakdown Voltage (kV)	11.4	18.6	11.8	12.8

**Table 2:** Numbering of four defect types.

Type	Metal Protrusion	Air Gap between the Metal Conductor and the Insulator	Pollution on the Insulator Surface	Charged Metal Particles
Number	1	2	3	4

**Table 3:** Confusion matrix of the RF classifier.

	Prediction	Type 1	Type 2	Type 3	Type 4
Actuality					
Type 1		8	0	0	0
Type 2		0	13	1	0
Type 3		2	0	5	0
Type 4		0	0	0	6

**Table 4:** Confusion matrix of the SVM classifier.

	Prediction	Type 1	Type 2	Type 3	Type 4
Actuality					
Type 1		7	1	0	0
Type 2		3	9	0	2
Type 3		0	0	6	1
Type 4		0	0	2	4

**Table 5:** Confusion matrix of the LR classifier.

	Prediction	Type 1	Type 2	Type 3	Type 4
Actuality					
Type 1		7	0	0	1
Type 2		0	14	0	0
Type 3		2	0	5	0
Type 4		1	0	0	5

## Data Availability

The data presented in this study may be available on request from the first author, Qipeng Tan. The data are not publicly available due to privacy reasons.
